# Engineering Chitosan-Coated PLGA Nanoparticles for Sustained Bupivacaine Delivery: Physicochemical Performance and a Preliminary Fibroblast Cytocompatibility Assessment

**DOI:** 10.3390/pharmaceutics18101325

**Published:** 2026-10-19

**Authors:** Gamze Çamlik, Tuba Kuşman Sayği, Kemal Alp Nalci

**Affiliations:** 1 Department of Pharmaceutical Technology, Faculty of Pharmacy, Biruni University, 34015 Istanbul, Türkiye; 2 Department of Pharmacognosy, Faculty of Pharmacy, Van Yüzüncü Yıl University, 65090 Van, Türkiye; tubakusman@yyu.edu.tr; 3 Department of Pharmacology, Faculty of Pharmacy, Van Yüzüncü Yıl University, 65090 Van, Türkiye; kemalalpnalci@yyu.edu.tr

**Keywords:** bupivacain hydrochloride, chitosan-coated PLGA nanoparticles, sustained drug release, local anesthetic delivery, fibroblast cytocompatibility, long-term stability

## Abstract

**Background/Objectives**: Bupivacaine hydrochloride (Bupi-HCl) is a widely used local anesthetic; however, rapid diffusion from the site of administration and concentration-dependent cytotoxicity may limit its local therapeutic efficacy. This study aimed to develop chitosan-coated poly(D, L-lactide-co-glycolide) (PLGA) nanoparticles for sustained Bupi-HCl delivery and to evaluate their physicochemical properties, release behavior, long-term stability, and cytocompatibility with fibroblasts. **Methods**: Bupi-HCl-loaded chitosan-coated PLGA nanoparticles (Bupi-CS-PLGA NPs) were prepared using a modified double-emulsion solvent-evaporation method and characterized by TEM, DLS, FTIR, DSC, XRD, encapsulation efficiency (EE), drug loading (DL), in vitro release, release kinetic modeling, 12-month physicochemical stability, and a preliminary MTT-based fibroblast cytocompatibility assessment. **Results**: Bupi-CS-PLGA NPs exhibited a predominantly spherical morphology, a mean particle size of 175.13 ± 8.10 nm, a PDI of 0.195 ± 0.026, and a zeta potential of +24.86 ± 1.90 mV. EE and DL were 80.00 ± 2.14% and 2.60 ± 0.11%, respectively. FTIR findings were consistent with physical incorporation of Bupi-HCl within the PLGA/chitosan matrix without evidence of new chemical bond formation, while DSC demonstrated differences in the thermal profiles of the nanoparticle formulations and XRD showed that Bupi-HCl was not detectable as a separate crystalline phase. The nanoparticles provided sustained Bupi-HCl release over 72 h, reaching approximately 79–80% cumulative release compared with 97–98% for free Bupi-HCl. Among the evaluated kinetic models, Modified Langmuir and RRSBW yielded the highest coefficients of determination (R^2^ = 0.9958 and 0.9952, respectively), while RRSBW showed the lowest total residual value (19.5851). Physicochemical characteristics were maintained during 12 months of storage at refrigerated and room temperatures. Within one experimental run, empty CS-PLGA NPs showed no decrease in MTT-derived signal across the tested concentration range. Bupi-CS-PLGA NPs showed lower MTT-derived signal at 250 and 500 µg/mL than at the two lower particle concentrations (62.5 and 125 µg/mL); particle-related assay interference was not excluded. Because free and encapsulated Bupi-HCl were not evaluated at dose-equivalent concentrations, their cytotoxic potency could not be directly compared, and these single-experiment findings are reported as a preliminary assessment. **Conclusions**: Bupi-CS-PLGA NPs provided sustained drug release and long-term physicochemical stability, together with a preliminary, assay-limited fibroblast MTT assessment. These findings support further evaluation of this biodegradable nanoparticle system for sustained local delivery of Bupi-HCl.

## 1. Introduction

Bupivacaine hydrochloride (Bupi-HCl) is a long-acting amide-type local anesthetic. Due to its prolonged sensory blockade and analgesic effect, Bupi-HCl is widely used in infiltration anesthesia, peripheral nerve blocks, epidural anesthesia, and spinal anesthesia [1,2]. However, conventional Bupi-HCl formulations have several limitations [3,4]. After local administration, the free drug may diffuse away from the site of administration, gradually reducing local drug concentration and duration of action [5]. Exposure to high concentrations of Bupi-HCl can induce concentration-dependent local cellular toxicity. It can also affect the viability of normal cells such as fibroblasts, which play a role in physiological tissue repair and wound healing [5,6]. Cardiovascular and central nervous system toxicity may also be associated with systemic absorption of Bupi-HCl [3]. These limitations highlight the need for safer and more controllable local drug delivery systems that can control drug exposure at the site of application [3,7].

Biodegradable polymeric nanoparticles offer a promising strategy for controlled delivery of local anesthetics [8]. Encapsulating a drug in a polymeric matrix can control release, reduce the rapid onset of exposure, and ensure long-term drug availability [3,9]. Depending on their composition and structural properties, polymeric nanoparticles can protect the encapsulated drug from environmental conditions and enhance its stability within the delivery system [9,10].

Poly(lactic-co-glycolic acid) (PLGA) is one of the most widely investigated biodegradable polymers for controlled drug-delivery applications because of its biocompatibility, tunable degradation behavior, and ability to provide sustained drug release through diffusion and polymer degradation [11,12]. Nevertheless, coating PLGA nanoparticles with natural polymers can further modify their surface properties and release behavior [13,14]. Chitosan is a biodegradable, biocompatible, and cationic polysaccharide containing protonatable amino groups [15,16]. Chitosan can coat PLGA nanoparticles to impart a positive charge, which may enhance their interaction with negatively charged biological surfaces [17]. More importantly, chitosan coating helps maintain colloidal stability and provides an additional diffusion barrier around the polymer matrix [16,18]. In the present study, chitosan coating was therefore employed primarily to modify the physicochemical surface characteristics and controlled-release behavior of the nanoparticles rather than to provide an independent therapeutic effect.

Researchers have previously investigated liposomal, microparticulate, and polymeric delivery systems to prolong the local action of bupivacaine [1]. Liposomal bupivacaine products have shown the feasibility of extending drug release in clinical practice; nevertheless, researchers continue to investigate alternative biodegradable polymeric carriers to expand sustained-release strategies for local anesthetic delivery [19,20].

Bupivacaine-loaded PLGA-based particulate systems have also been investigated previously, demonstrating the feasibility of biodegradable polymeric carriers for modulating bupivacaine release and prolonging local anesthetic effects [21,22]. Chitosan-containing nanocarriers have likewise been explored for bupivacaine delivery, including alginate/chitosan nanoparticles evaluated in terms of drug release, cytocompatibility, and local anesthetic efficacy [23,24]. These studies demonstrate that both PLGA-based and chitosan-containing carrier strategies for bupivacaine delivery have been explored previously. However, these systems differ from the present Bupi-CS-PLGA NPs in carrier architecture and/or in the scope of physicochemical, stability, release, and biological characterization. Although a related chitosan-coated PLGA system carrying benzocaine has been evaluated in 3T3 fibroblasts and HaCaT keratinocytes [25], evidence specifically addressing Bupi-CS-PLGA NPs in primary adult human dermal fibroblasts remains limited. This is especially important because fibroblasts are involved in extracellular matrix and collagen synthesis, as well as tissue remodeling, but high concentrations of local anesthetics can adversely affect their viability [26]. Furthermore, local anesthetic cellular toxicity has been linked to oxidative stress; thus, biological safety must be evaluated alongside pharmaceutical performance [26,27]. Therefore, fibroblast cytocompatibility was evaluated as a preliminary and complementary biological parameter using the MTT assay, while the DPPH assay was included as an exploratory chemical radical-scavenging assessment. Because the DPPH assay reflects chemical radical-scavenging capacity rather than cellular oxidative stress, it was interpreted only as complementary physicochemical information.

This study aimed to provide an integrated evaluation of the physicochemical and biological performance of Bupi-HCl-loaded chitosan-coated PLGA nanoparticles. Bupi-CS-PLGA NPs were characterized for morphology, particle size, polydispersity index, zeta potential, chemical structure, thermal behavior, crystallinity, encapsulation efficiency, and drug-loading capacity. Their sustained-release behavior, long-term physicochemical stability, release kinetics, preliminary fibroblast cytocompatibility, and an exploratory DPPH radical-scavenging response were also investigated. Accordingly, the novelty of the present study does not rely on the use of PLGA or chitosan alone, but on the integrated evaluation of Bupi-HCl within a chitosan-coated PLGA nanoparticle system, combining detailed physicochemical and solid-state characterization, sustained-release assessment and kinetic modeling, 12-month physicochemical stability, and preliminary cytocompatibility evaluation in primary adult human dermal fibroblasts.

## 2. Materials and Methods

### 2.1. Materials

Bupivacaine hydrochloride (Sigma-Aldrich, St. Louis, MO, USA), poly(D,L-lactide-co-glycolide) (PLGA; lactide: glycolide ratio 75:25; Sigma-Aldrich, St. Louis, MO, USA), low-molecular-weight chitosan (Sigma-Aldrich, St. Louis, MO, USA), poly(vinyl alcohol) (PVA; Merck, Darmstadt, Germany), Span^®^ 80 (Sigma-Aldrich, St. Louis, MO, USA), caprylic/capric triglyceride (Merck, Darmstadt, Germany), ethyl acetate (Merck, Darmstadt, Germany), glacial acetic acid (Merck, Darmstadt, Germany), 2,2-diphenyl-1-picrylhydrazyl (DPPH; Sigma-Aldrich, St. Louis, MO, USA), L-ascorbic acid (Sigma-Aldrich, St. Louis, MO, USA), and butylated hydroxytoluene (BHT; Sigma-Aldrich, St. Louis, MO, USA) were used in this study. For in vitro release studies, a pH 7.4 buffer solution was prepared using phosphate-buffered saline (PBS) tablets (BioBasic Inc., Markham, ON, Canada).

Normal human adult dermal fibroblasts (HDFa; ATCC PCS-201-012; American Type Culture Collection, Manassas, VA, USA) were used in cell culture studies. The cells were cultured in medium containing high-glucose Dulbecco’s Modified Eagle Medium (DMEM; Capricorn Scientific, Ebsdorfergrund, Germany), 10% fetal bovine serum (FBS; Biowest, Nuaillé, France), and 1% penicillin–streptomycin–amphotericin B solution (Gibco, Thermo Fisher Scientific, Waltham, MA, USA). In cell passage, 0.25% trypsin–EDTA solution (Gibco, Thermo Fisher Scientific, Waltham, MA, USA) was used; Trypan Blue solution (Gibco, Thermo Fisher Scientific, Waltham, MA, USA) was used to determine cell viability; 3-(4,5-dimethylthiazol-2-yl)-2,5-diphenyltetrazolium bromide (MTT; Sigma-Aldrich, St. Louis, MO, USA) was used for cytotoxicity analysis; and dimethyl sulfoxide (DMSO; Sigma-Aldrich, St. Louis, MO, USA) was used to dissolve the resulting formazan crystals. All chemicals used in the study were of analytical purity and were used without further purification.

### 2.2. Preparation of Bupivacaine HCl-Loaded Chitosan-Coated PLGA Nanoparticles (Bupi-CS-PLGA NPs)

Bupi-CS-PLGA NPs were prepared by solvent evaporation using a modified double emulsion (water/oil/water, *w*/*o*/*w*) method [28]. To prepare the organic phase, 30 mg of PLGA was completely dissolved in 0.9 mL of ethyl acetate. Then, 40 µL of caprylic/capric triglyceride (CCT) and 15 µL of Span^®^ 80 were added to obtain a homogeneous organic phase. The Bupi-HCl stock solution was prepared by dissolving 10 mg of the active substance in 1 mL of distilled water. An amount of 100 µL of this stock solution was taken to obtain the inner aqueous phase containing 1 mg of Bupi-HCl. The inner aqueous phase was slowly added to the organic phase under continuous magnetic stirring at 1200 rpm to form the primary water/oil (*w*/*o*) pre-emulsion. Probe sonication was not applied at this stage due to the low volume of the primary emulsion and the fact that the probe geometry used was not suitable for effective and repeatable sonication in this volume.

The prepared primary emulsion was slowly added dropwise into the external aqueous phase containing 0.25 g of PVA, while the mixture was continuously stirred at 1000 rpm using a magnetic stirrer. Stirring continued for 1 additional minute after the dropwise addition was complete. The resulting double emulsion was sonicated for 50 s in pulsed mode (5 s on and 5 s off) at 50% amplitude using a SONOPULS HD 4050 ultrasonic homogenizer (BANDELIN electronic GmbH & Co. KG, Berlin, Germany), while the sample was maintained in an ice bath to minimize temperature elevation during sonication. The double emulsion was stirred with a magnetic stirrer at room temperature for 24 h to evaporate the ethyl acetate. After the solvent was evaporated, 0.01% (*w*/*v*) chitosan in 0.1% (*w*/*v*) acetic acid was added to the nanoparticle dispersion. The dispersion was stirred with a magnetic stirrer for 1 h to coat the nanoparticles with chitosan.

After the coating process, the nanoparticles were centrifuged. The centrifuged samples were washed three times with distilled water. This was done to eliminate the free drug, unadsorbed chitosan, and residual surfactants. The resulting nanoparticle dispersion was directly used for particle size, polydispersity index (PDI), zeta potential, encapsulation efficiency, in vitro drug release studies, and biological evaluations. For DSC and XRD analyses, only the portion of the nanoparticle dispersion intended for solid-state characterization was lyophilized (Alpha 1–2 LDplus, Martin Christ, Osterode am Harz, Germany).

### 2.3. Physicochemical Characterization

#### 2.3.1. Physical Appearance, Particle Size, Polydispersity Index (PDI), and Zeta Potential

To measure the size and morphology of Bupi-CS-PLGA NPs, TEM (Carl Zeiss EM 900, Jena, Germany) was employed. The particle size, PDI, and zeta potential were assessed using an Anton Paar LiteSizer 500. Ten replicate measurements were performed on samples obtained from the same prepared formulation (*n* = 10), and the results were expressed as mean ± SD.

#### 2.3.2. Fourier Transform Infrared Spectroscopy (FTIR)

Fourier transform infrared spectroscopy (FTIR) analyses (INVENIO S FT-IR Spectrometer, Bruker Optics GmbH, Ettlingen, Germany) were performed to determine possible chemical interactions between Bupi-HCl, PLGA, chitosan, empty nanoparticles, and Bupi-CS-PLGA NPs. The analyses were carried out in the wavelength range of 4000–400 cm^−1^ on an FTIR spectrometer equipped with an ATR unit. Prior to the analysis, the background spectrum was acquired, and baseline correction was applied to all spectra. The obtained spectra were compared among pure Bupi-HCl, PLGA, and chitosan, as well as empty and drug-loaded nanoparticles, to evaluate characteristic functional groups, band shifts, and the formation of new absorption bands.

#### 2.3.3. Differential Scanning Calorimetry (DSC)

Differential scanning calorimetry (DSC) was performed using a DSC7000X instrument (Hitachi High-Tech Science Corporation, Tokyo, Japan) to evaluate the thermal behavior of the formulation components and nanoparticle formulations. PLGA, chitosan, empty CS-PLGA NPs, and Bupi-CS-PLGA NPs were analyzed separately. Approximately 5–7 mg of each sample was placed in aluminum pans, and an empty aluminum pan was used as the reference. Samples were heated from 25 to 330 °C at a constant heating rate of 10 °C/min under a nitrogen atmosphere. The resulting thermograms were evaluated for glass-transition behavior, endothermic and exothermic thermal events, peak shifts, and overall changes in thermal profiles.

#### 2.3.4. X-Ray Diffraction (XRD)

Using X-ray diffraction (Rigaku MiniFlex 600 Powder X-ray Diffractometer, Tokyo, Japan), we analyzed the crystal structures of unmodified Bupi-HCl, PLGA, and chitosan, as well as empty and Bupi-CS-PLGA NPs. The XRD was performed with CuKα radiation (λ = 1.5406 Å) at 40 kV and 15 mA. Diffractograms were obtained for the 5–60° (2θ) range with a 0.02° step width. Diffraction patterns were compared to evaluate differences in crystallinity between the pure components and the nanoparticle formulations. The major peaks of diffraction were found on the basis of their 2θ value, and special focus was laid upon the identification of Bupi-HCl’s distinctive crystalline peaks after embedding in the CS-PLGA nanoparticles.

#### 2.3.5. Encapsulation Efficiency (EE) and Drug Loading (DL)

Encapsulation efficiency (EE) was determined indirectly by quantifying the amount of free Bupi-HCl in the supernatant after centrifugation at 15,000 rpm for 15 min. Bupi-HCl concentrations were determined by UV–visible spectrophotometry at 201 nm using a UV–visible spectrophotometer (Hinotek, Ningbo, China). For quantitative analysis, a 100 µg/mL Bupi-HCl stock solution was prepared, from which standard solutions at concentrations of 2, 4, 6, 8, 10, 15, and 20 µg/mL were obtained by appropriate dilution.

The calibration curve was linear in the investigated concentration range with regression equation of y = 0.0395x − 0.0453 and R^2^ = 0.999. Encapsulated Bupi-HCl (We) was calculated by subtracting free Bupi-HCl (Wf) detected in the supernatant from the total Bupi-HCl (Wt) initially added to the formulation.

EE was calculated using the following equation [29]:

$$EE\left(\%\right)=\frac{Wt-Wf}{Wt}\;\times 100$$

The DL was calculated using the following equation [30]:

$$DL\left(\%\right)=\frac{We}{Wm}\;\times 100$$

where W_t_ represents the total amount of Bupi-HCl initially added to the formulation, W_f_ represents the amount of free (non-encapsulated) Bupi-HCl, We represents the amount of encapsulated Bupi-HCl, and W_m_ represents the dry mass of the recovered drug-loaded nanoparticles. For determination of W_m_, the nanoparticle pellet obtained after centrifugation was dried in an oven until a constant mass was achieved and subsequently weighed.

EE and DL were determined from three analytical replicate measurements performed on samples obtained from the same prepared formulation (n = 3), and the results were expressed as mean ± SD.

### 2.4. In Vitro Drug Release Study

The in vitro release profile of Bupi-HCl from Bupi-CS-PLGA NPs was evaluated using Franz diffusion cells equipped with semipermeable cellophane membranes. Before use, the cellophane membranes were hydrated in PBS (pH 7.4) and subsequently mounted between the donor and receptor compartments. The receptor compartment (7.5 mL) was filled with PBS (pH 7.4) and maintained at 37 ± 0.5 °C throughout the experiment [31]. The effective diffusion area of the Franz diffusion cells was 1.77 cm^2^.

A 1 mL aliquot of the Bupi-CS-PLGA NP dispersion was placed in the donor compartment. A free Bupi-HCl solution was used as the control. The amount of Bupi-HCl initially placed in the donor compartment was equivalent in the free Bupi-HCl and Bupi-CS-PLGA NP groups to allow comparison of their release profiles. The release experiments were performed in triplicate (n = 3), and the results were expressed as mean ± standard deviation (SD). At predetermined sampling times (0.5, 1, 2, 4, 8, 12, 24, 36, and 72 h), 2 mL of receptor medium was withdrawn and immediately replaced with an equal volume of fresh PBS. Bupi-HCl concentrations in the collected samples were determined by UV–visible spectrophotometry at 201 nm using the previously established calibration curve. Cumulative drug release was expressed as a percentage of the initial Bupi-HCl amount. The cumulative amount released at each sampling point was corrected for the Bupi-HCl removed during previous sampling and for replacement of the receptor medium with fresh PBS.

To mathematically characterize the release profile, cumulative Bupi-HCl release data obtained from Bupi-CS-PLGA NPs were fitted to zero-order, first-order, Higuchi, Hixson–Crowell, Hopfenberg, Langmuir, Modified Langmuir, BTa, and RRSBW models using In Vitro–In Vivo Kinetic Correlation and Routine Software (Version 1.0.40, Faculty of Pharmacy, Ege University, Izmir, Türkiye) [32]. Zero-order and first-order models describe concentration-independent and concentration-dependent release behavior, respectively, whereas the Higuchi model describes release as a function of the square root of time and is commonly applied to evaluate diffusion-related release from matrix systems [33,34]. The Hixson–Crowell model considers changes in particle geometry during dissolution, whereas the Hopfenberg model describes release associated with surface erosion [35]. The Rosin–Rammler–Sperling–Bennett–Weibull (RRSBW) model is an empirical distribution model that has been applied for the mathematical characterization and comparison of pharmaceutical dissolution and release profiles [36]. The model includes parameters describing the characteristic time/scale and the shape of the release profile. The (*Bt*)*^a^* (BTa) model has likewise been used in pharmaceutical dissolution and release-kinetic analyses as an empirical mathematical model [37]. In the present study, these models were used for comparative mathematical characterization of the experimental release profile rather than for assignment of a specific physicochemical release mechanism. The coefficient of determination (R^2^) and the total residual value provided by the computer software were used as supplementary methods for assessing the mathematical compatibility between the actual release data and the model curves. The kinetic modeling was conducted as a mathematical comparison of the release profile data, and not as conclusive proof of the release mechanism.

### 2.5. Cell Culture and Cytocompatibility Evaluation

#### 2.5.1. Cell Culture

Normal human adult dermal fibroblasts (HDFa; ATCC PCS-201-012) were cultured in high-glucose Dulbecco’s Modified Eagle Medium (DMEM; Capricorn Scientific, Ebsdorfergrund, Germany) supplemented with 10% fetal bovine serum (FBS; Biowest, Nuaillé, France) and 1% penicillin–streptomycin–amphotericin B solution (Gibco, Thermo Fisher Scientific, Waltham, MA, USA). Cells were maintained at 37 °C in a humidified atmosphere containing 5% CO_2_. The culture medium was renewed every 2–3 days, and cells were subcultured at approximately 80% confluence using 0.25% trypsin–EDTA. Only cells in the logarithmic growth phase were used in the experiments. Cell viability before seeding was assessed using the trypan blue exclusion method and was >95%. The experiments were conducted using cells at passage 24.

#### 2.5.2. Test Formulations and Concentration Convention

Free Bupi-HCl, empty chitosan-coated PLGA nanoparticles (CS-PLGA NPs), and Bupi-HCl-loaded chitosan-coated PLGA nanoparticles (Bupi-CS-PLGA NPs) were evaluated. Concentrations of free Bupi-HCl were expressed as drug mass per unit volume, whereas concentrations of the nanoparticle formulations were expressed as total particle mass per unit volume.

Based on the experimentally determined drug-loading capacity of 2.60% (*w*/*w*), Bupi-CS-PLGA NP concentrations of 62.5, 125, 250, and 500 µg/mL corresponded to nominal Bupi-HCl-equivalent concentrations of 1.63, 3.25, 6.50, and 13.0 µg/mL, respectively. Nanoparticle concentrations were expressed as total particle mass per unit volume, whereas the corresponding Bupi-HCl-equivalent concentrations represented the nominal amount of encapsulated drug calculated from the measured drug-loading capacity. Therefore, free Bupi-HCl and nanoparticle concentration scales were not considered directly interchangeable. Accordingly, direct dose-equivalent comparisons between free Bupi-HCl and Bupi-CS-PLGA NPs were not performed. The nominal concentrations reported denote the final in-well concentrations after 1:1 addition to the medium already present in each well. Nanoparticle mass concentrations were derived from the dry particle mass of the washed dispersion, free Bupi-HCl was prepared in complete culture medium, and all working dispersions were prepared under aseptic conditions immediately before use.

#### 2.5.3. MTT Cytocompatibility Assay

The effects of free Bupi-HCl, empty CS-PLGA NPs, and Bupi-CS-PLGA NPs on HDFa cell viability were evaluated using the MTT [3-(4,5-dimethylthiazol-2-yl)-2,5-diphenyltetrazolium bromide] colorimetric assay [38,39]. HDFa cells were seeded into 96-well plates at a density of 4 × 10^3^ cells per well in 100 µL of complete culture medium and allowed to adhere for 24 h. Peripheral wells were filled with sterile Dulbecco’s phosphate-buffered saline (DPBS; Gibco, Thermo Fisher Scientific, Waltham, MA, USA) to minimize evaporation-related edge effects, and only the inner wells were used for experimental treatments.

After the adhesion period, cells were exposed to free Bupi-HCl or empty CS-PLGA NPs at nominal concentrations of 31.25, 62.5, 125, 250, and 500 µg/mL. Bupi-CS-PLGA NPs were tested at nominal particle concentrations of 62.5, 125, 250, and 500 µg/mL. The loaded formulation was not evaluated at 31.25 µg/mL. Treatments were prepared by two-fold serial dilution of 2× working dispersions in complete culture medium. Subsequently, 100 µL of each working dispersion was added to the 100 µL of medium already present in each well.

Untreated cells were used as the negative control and represented 100% viability. Cell-free wells containing culture medium alone were used as plate-specific optical blanks. For each plate, the mean blank absorbance was used for blank correction; the single optical-blank exclusion on the free-drug plate is described in Section 2.8. Cell-free wells containing nanoparticle dispersions were not included. Nanoparticle dispersions were vortexed immediately before application to the cells. Free Bupi-HCl and the nanoparticle formulations were tested on separate plates, each containing its own control and blank wells. Each treatment condition was evaluated in six technical replicate wells within one independent experiment.

Following 48 h of exposure at 37 °C and 5% CO_2_, the treatment medium was removed and replaced with 90 µL of fresh complete medium and 10 µL of MTT stock solution prepared at 5 mg/mL in PBS (Gibco, Thermo Fisher Scientific, Waltham, MA, USA). The final MTT concentration in each well was 0.5 mg/mL. Plates were incubated for an additional 4 h to allow formazan crystal formation. The supernatant was subsequently removed, and 100 µL of dimethyl sulfoxide was added to each well to dissolve the formazan crystals [38,39]. Plates were gently agitated for 2 min and incubated in the dark for a further 10 min. Absorbance was measured at 570 nm using a FLUOstar Omega microplate reader (BMG LABTECH GmbH, Ortenberg, Germany).

Cell viability was calculated using the following equation:

Viability (%) = [(*A*_sample_ − *A*_blank_)/(*A*_control_ − *A*_blank_)] × 100

where *A*_sample_, *A*_blank_, and *A*_control_ represent the absorbance of the treated cells, optical blank, and untreated control cells, respectively.

### 2.6. Stability Study

The prepared Bupi-CS-PLGA NP formulation was subjected to stability testing for 12 months under refrigerated conditions (5 ± 2 °C) and at 25 ± 2 °C/60 ± 5% relative humidity. Particle size, PDI, and zeta potential were evaluated at 0, 6, and 12 months. At each time point, 10 replicate measurements were performed on samples obtained from the same stored formulation (n = 10), and the results were expressed as mean ± SD. Measurements were performed using a LiteSizer 500 (Anton Paar, Graz, Austria).

### 2.7. DPPH Assay

Free Bupi-HCl, empty CS-PLGA NPs, and Bupi-CS-PLGA NPs were examined using a microplate adaptation of the DPPH assay described by Blois [40]. Ascorbic acid and butylated hydroxytoluene (BHT) were used as reference antioxidants. A 0.1 mM DPPH solution was freshly prepared in absolute ethanol and protected from light. Free Bupi-HCl and nanoparticle formulations were prepared in ultrapure water and serially diluted to concentrations of 10, 25, 50, 125, 250, 500, and 1000 µg/mL with ethanol.

Aliquots of 150 µL of each sample or reference antioxidant solution were mixed with 50 µL of DPPH solution in a 96-well microplate. Control wells contained 150 µL ethanol and 50 µL DPPH solution. Solvent-only blanks contained neither DPPH nor test sample. Separate sample blanks without DPPH were not prepared. The results are reported as raw absorbance at 517 nm without solvent- or sample-blank subtraction.

The reaction mixtures were incubated for 30 min at 25 ± 2 °C in the dark, and absorbance was measured at 517 nm using a microplate reader (FLUOstar Omega, BMG LABTECH, Ortenberg, Germany). All measurements were performed in triplicate (n = 3).

For each sample and reference antioxidant concentration, the arithmetic mean and sample standard deviation were calculated from the three individual raw absorbance readings at 517 nm (n = 3). No solvent-blank or sample-blank subtraction was applied to the reported values. The mean control absorbance was calculated from the 21 DPPH-plus-solvent control wells. Results are presented as raw absorbance rather than percentage DPPH scavenging activity because separate sample blanks without DPPH were not measured.

### 2.8. Statistical Analysis

Unless otherwise stated, quantitative data were expressed as mean ± standard deviation (SD). Particle size, PDI, zeta potential, encapsulation efficiency (EE), drug loading (DL), and stability data were summarized descriptively as replicate measurements obtained from the same prepared formulation; no inferential statistical comparisons were performed for these datasets. Statistical analyses for the MTT assay were performed using IBM SPSS Statistics, version 25.0 (IBM Corp., Armonk, NY, USA). Normality of the MTT data was assessed using the Shapiro–Wilk test, and homogeneity of variances was assessed using the median-centered Levene (Brown–Forsythe) test. Because homogeneity of variance could not be assumed across all MTT groups, each treatment concentration was compared with the corresponding untreated control from the same plate using Welch’s *t*-test. The resulting MTT *p*-values were adjusted for multiple comparisons using the Holm method [41].

Empty CS-PLGA NPs and Bupi-CS-PLGA NPs were additionally compared at matched nominal particle concentrations using Welch’s *t*-test with Holm adjustment. On the free-drug plate, Grubbs’ test (α = 0.05) [42] identified one cell-free optical-blank well (OD570 = 0.058) as an outlier (G = 4.823; critical value = 2.991). This well was excluded only from calculation of the plate-specific blank; the remaining 35 optical-blank wells yielded a mean OD570 of 0.035, and the original reading was retained in the source dataset. The same test was applied as an exploratory screen to the six technical treatment wells within each group, and no treatment wells were excluded from the primary analysis. One empty CS-PLGA well at 500 µg/mL was flagged only marginally (G = 1.901; critical value 1.887) and was retained (100.2 ± 13.8%, *n* = 6); excluding it as a sensitivity analysis gave 105.5 ± 5.6% (*n* = 5) without altering any conclusion. Statistical significance was set at *p* < 0.05. Where applicable, significance levels are indicated as *p < 0.05, **p < 0.01, and ***p < 0.001. MTT data are presented as mean ± SD of six technical replicate wells from a single independent experiment. Because the six wells per condition are technical rather than biological replicates, the statistical comparisons represent within-run exploratory comparisons based on technical replicate wells; they may indicate differences within a single experimental run but do not establish biological reproducibility. Independent biological replication is required for confirmatory inference, and the cytocompatibility findings are therefore presented as preliminary rather than confirmatory.

## 3. Results

### 3.1. Physicochemical Characterization

Physical Appearance, Particle Size, Polydispersity Index (PDI), and Zeta Potential.

**Figure 1:**
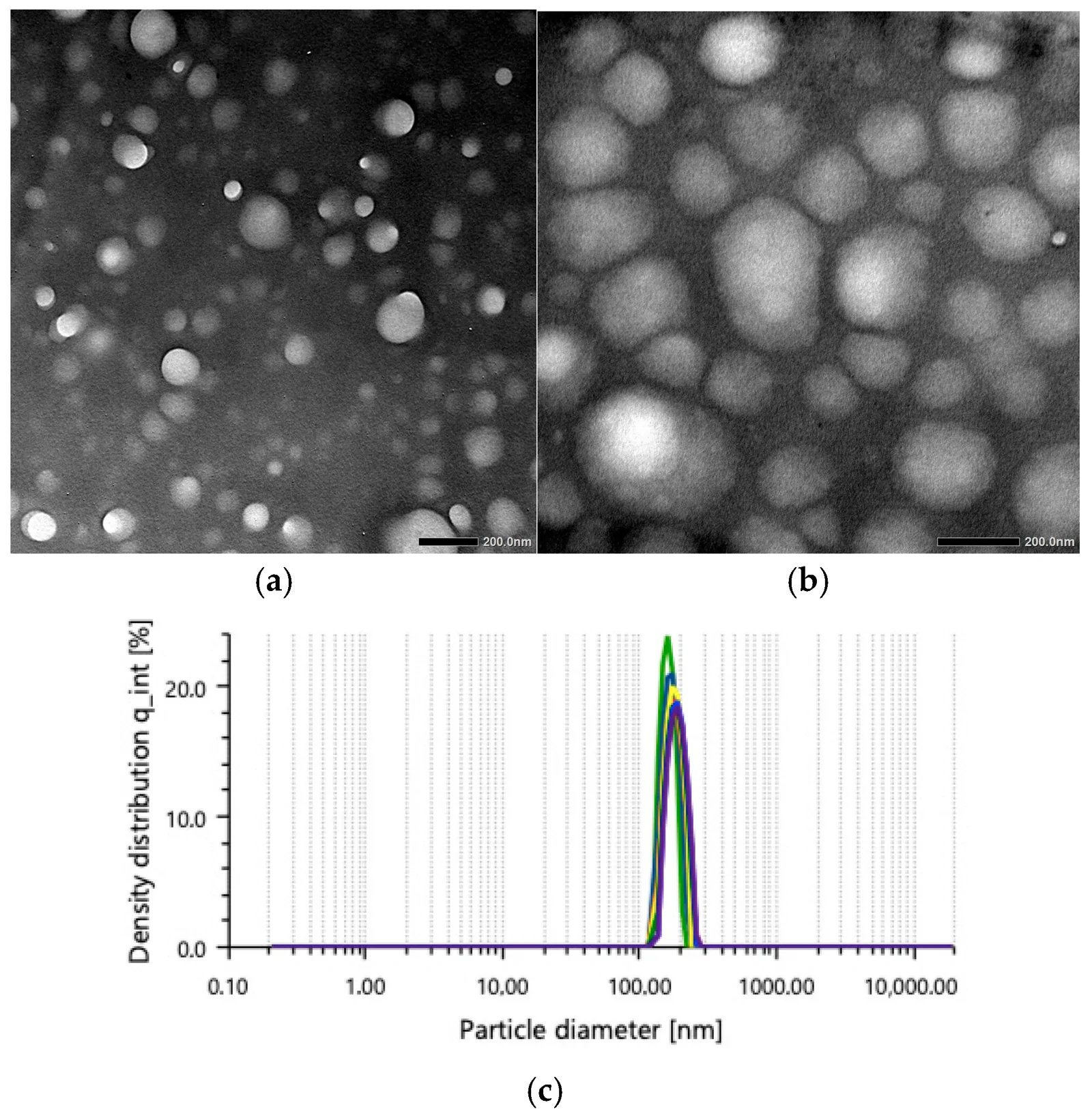
Morphological and particle-size characterization of the nanoparticles: TEM images of (**a**) empty CS-PLGA NPs and (**b**) Bupi-CS-PLGA NPs, and (**c**) intensity-weighted DLS particle-size distribution profiles of Bupi-CS-PLGA NPs.

The prepared Bupi-CS-PLGA NP dispersions appeared uniform and opaque white, with no visible precipitation, aggregation, or phase separation. The morphology of empty CS-PLGA NPs and Bupi-CS-PLGA NPs was evaluated by TEM (Figure 1a,b). Both formulations exhibited predominantly spherical or near-spherical particles with relatively smooth surfaces. The particles were generally well dispersed, although limited particle contact was observed in some areas.

For dynamic light scattering (DLS) analysis, particle size, polydispersity index (PDI), and zeta potential were measured. The mean hydrodynamic particle size of CS-PLGA NPs was determined to be 161.150 ± 5.601 nm, the PDI value 0.148 ± 0.012, and the zeta potential +24.104 ± 1.021 mV. After Bupi-HCl loading, the mean particle size increased to 175.132 ± 8.103 nm, while the PDI value became 0.195 ± 0.026 and the zeta potential was measured as +24.861 ± 1.90 mV. The intensity-weighted DLS particle-size distribution profiles of Bupi-CS-PLGA NPs are presented in Figure 1c, complementing the numerical hydrodynamic particle-size and PDI data reported in Table 1.

#### 3.1.1. Fourier Transform Infrared Spectroscopy

**Figure 2:**
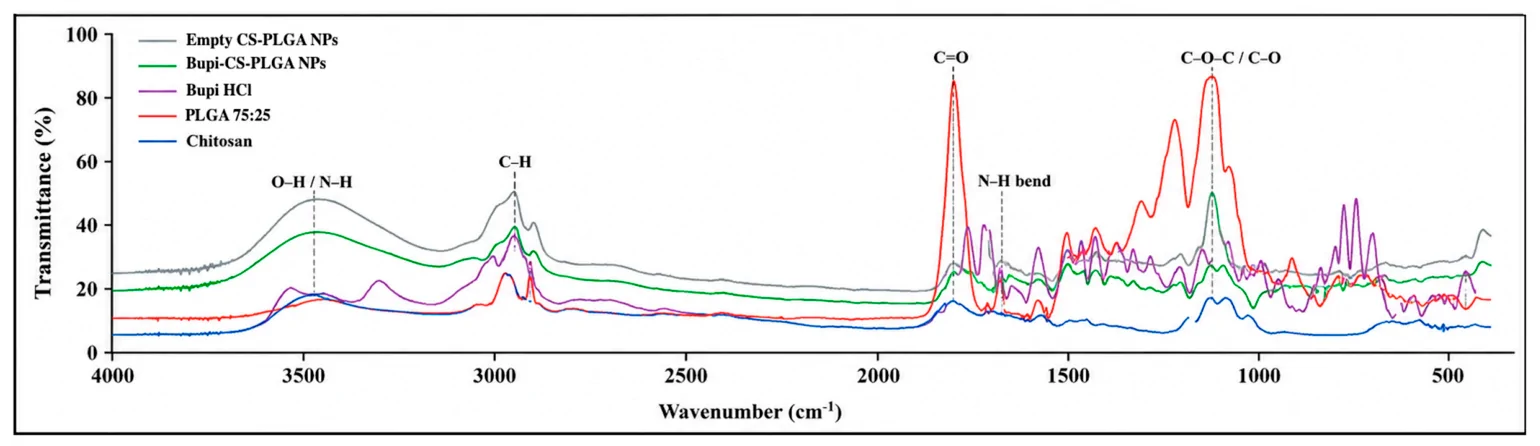
FTIR spectra of empty CS-PLGA NPs, Bupi-CS-PLGA NPs, chitosan, PLGA 75:25, and Bupi-HCl.

The FTIR spectra of Bupi-HCl, PLGA, chitosan, empty CS-PLGA NPs, and Bupi-CS-PLGA NPs are given in Figure 2.

In the pure Bupi-HCl spectrum, characteristic bands were observed corresponding to N–H stretching vibrations in the approximately 3450 cm^−1^ region, aliphatic C–H stretching bands in the 2950–2850 cm^−1^ range, aromatic ring and N–H bending vibrations in the 1600–1650 cm^−1^ region, and C–N and C–O vibrations in the 1300–1000 cm^−1^ range. Additionally, a characteristic absorption band belonging to the C–Cl group was identified at low wavenumbers.

In the pure PLGA spectrum, a strong characteristic peak belonging to the ester carbonyl (C=O) group was observed at approximately 1750 cm^−1^, and C–O–C stretching vibrations belonging to the ester structure were clearly observed in the range of 1180–1080 cm^−1^.

In the chitosan spectrum, a broad O–H/N–H stretching band belonging to the hydroxyl and amino groups was detected in the range of 3200–3500 cm^−1^, characteristic absorption bands belonging to the amino and amide groups were observed in the region of 1590–1650 cm^−1^, and C–H stretching vibrations were detected around 2900 cm^−1^.

In the FTIR spectrum of empty CS-PLGA NPs, both the carbonyl band belonging to PLGA and the hydroxyl/amino bands belonging to chitosan were observed to be conserved. In CS-PLGA nanoparticles loaded with Bupi-HCl, the characteristic bands of PLGA and chitosan were preserved, while the intensity of some characteristic Bupi-HCl peaks decreased and some bands showed slight wavenumber shifts. However, it was determined that no new absorption band formed in the formulation.

#### 3.1.2. Differential Scanning Calorimetry

**Figure 3:**
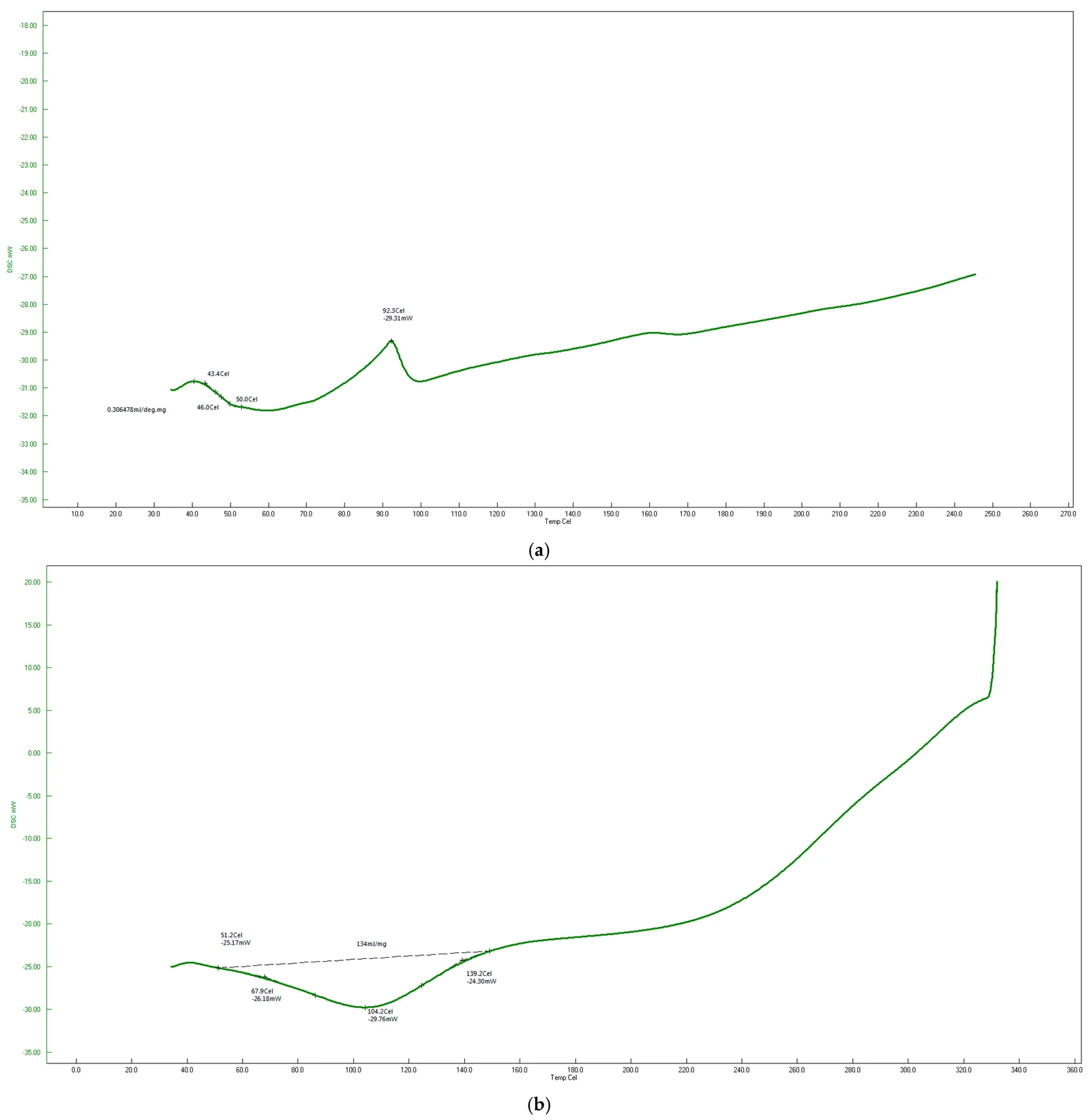

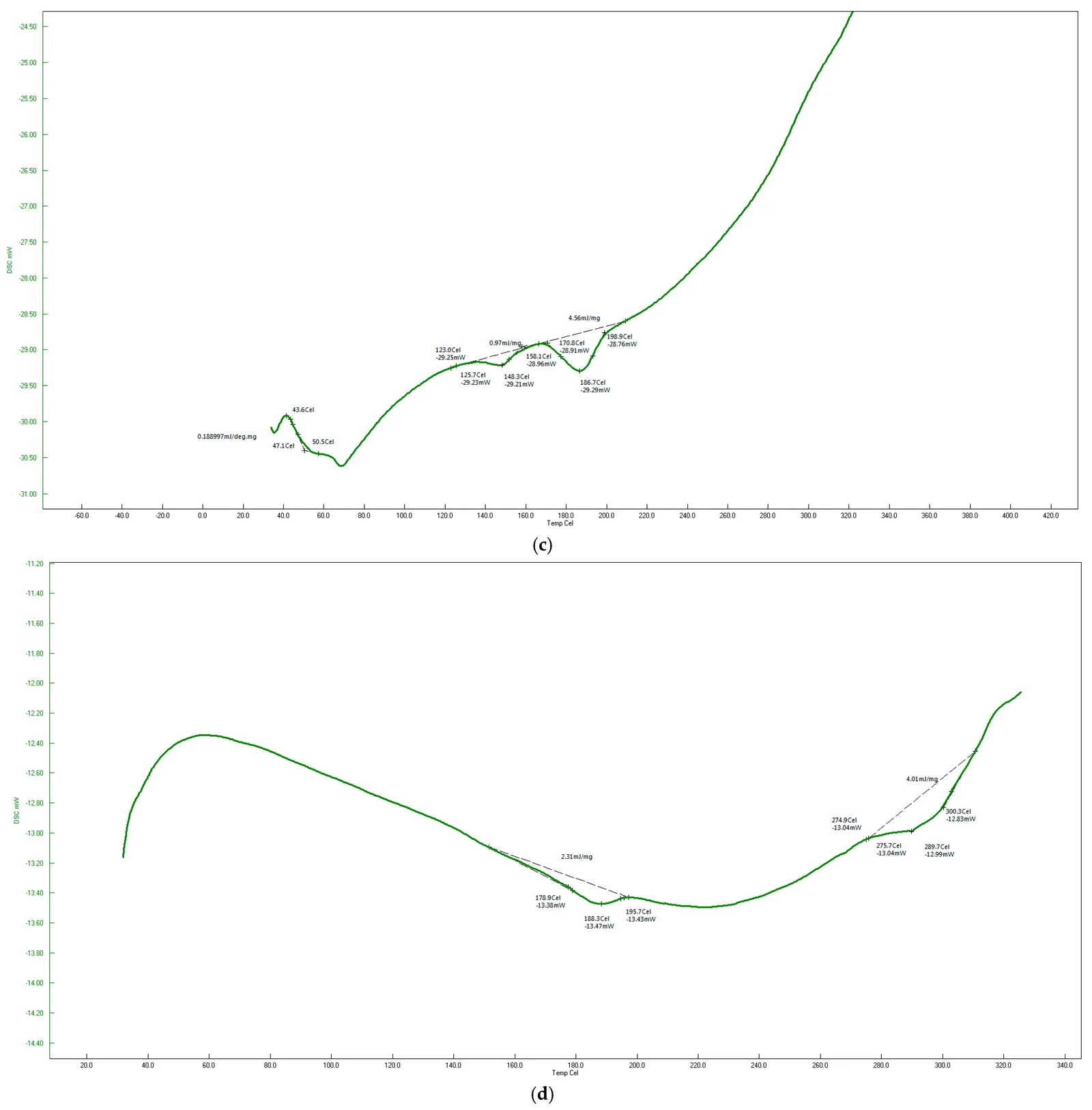
Differential scanning calorimetry (DSC) thermograms of PLGA (**a**); Chitosan (**b**); empty CS-PLGA NPs (**c**), and Bupi-CS-PLGA NPs (**d**).

DSC thermograms of PLGA 75:25, chitosan, empty CS-PLGA NPs, and Bupi-CS-PLGA NPs are shown in Figure 3.

Thermogram of PLGA 75:25 showed a thermal transition between 43.4 and 50.5 °C range, which was in accordance with the glass-transition behavior of the amorphous polymer, as well as an endothermic transition peak at 92.3 °C. No distinct melting endotherm was observed, consistent with the predominantly amorphous nature of PLGA. The chitosan thermogram exhibited low-temperature endothermic features in the approximately 44.9–104.2 °C region, which may be associated with the loss of absorbed and bound water. A high-temperature thermal event was observed near 330 °C.

The nanoparticle formulations exhibited thermal profiles that differed from those of the individual polymeric components. Empty CS-PLGA NPs showed several thermal features over the investigated temperature range, while the Bupi-CS-PLGA NP thermogram exhibited changes in the position, intensity, and breadth of the observed thermal events. In particular, broader and less distinct thermal features were observed in the drug-loaded nanoparticle thermogram compared with the empty nanoparticles. Overall, changes in the thermal profiles were observed following nanoparticle formation and Bupi-HCl incorporation. Because pure Bupi-HCl was not analyzed separately by DSC, the thermal events observed in the Bupi-CS-PLGA NP thermogram were not specifically assigned to Bupi-HCl.

#### 3.1.3. X-Ray Diffraction

**Figure 4:**
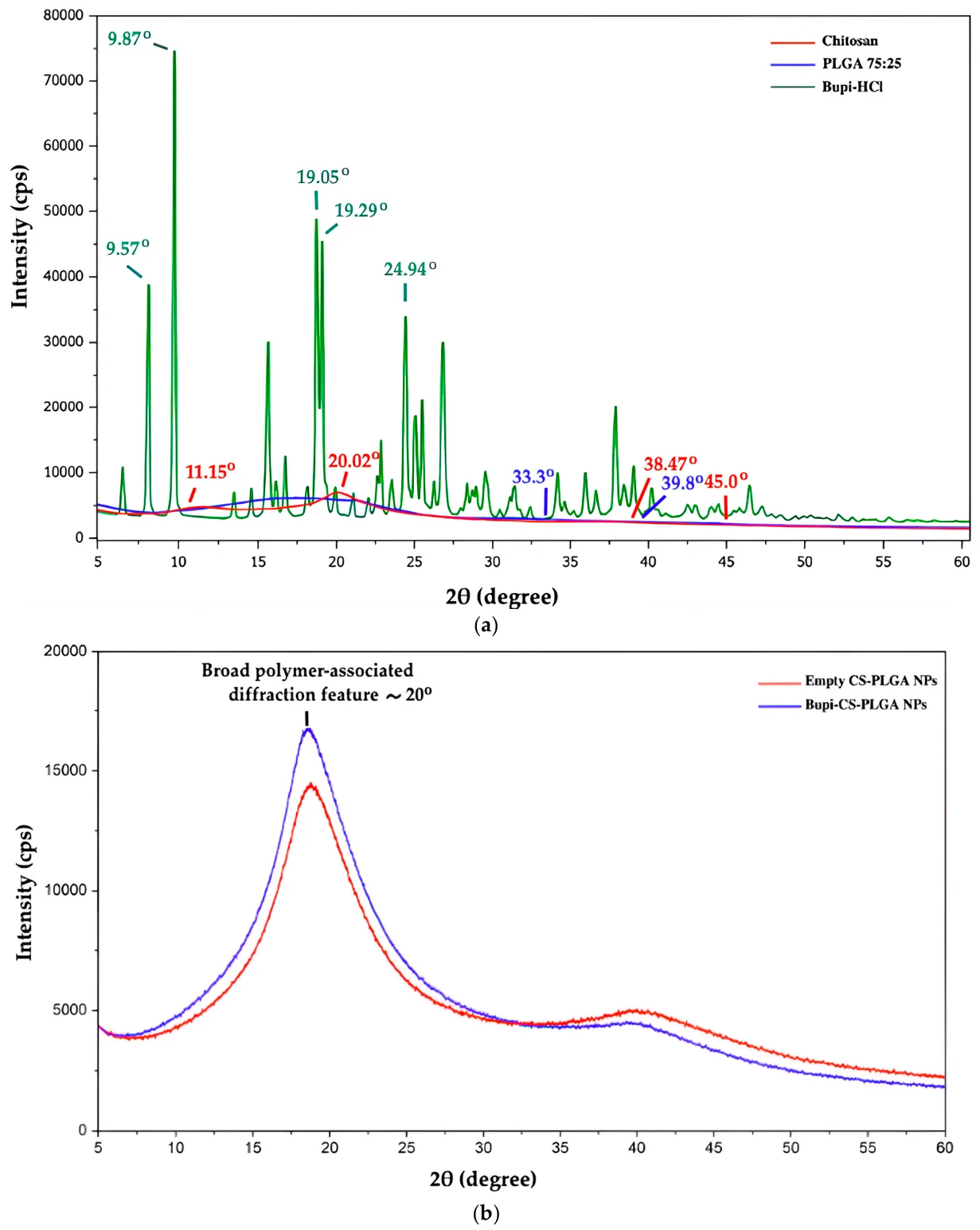
X-ray diffraction (XRD) patterns of (**a**) chitosan, PLGA (75:25), and pure Bupi-HCl and (**b**) empty CS-PLGA NPs and Bupi-CS-PLGA NPs. Major diffraction features are labeled with their corresponding experimental 2θ values.

The X-ray diffraction (XRD) was conducted to study the solid-state properties of Bupi-HCl, PLGA (75:25), chitosan, empty CS-PLGA nanoparticles, and Bupi-CS-PLGA nanoparticles (Figure 4). The crystalline nature of pure Bupi-HCl was revealed from the presence of several peaks showing sharp intensity. The main peaks in the experiment were recorded at 2θ = 9.57°, 9.87°, 19.05°, 19.29°, and 24.94°. The results were found to be in accordance with the reported crystalline diffraction pattern of Bupi-HCl in the literature [43]. Chitosan showed typical peaks at 2θ = 11.15° and 20.02°, along with relatively weak peaks at 38.47° and 45.0°. The typical peak found around 20° was in agreement with the typical diffraction range reported for chitosan [44]. The PLGA presented a broad diffraction pattern in accordance with its amorphous state, with weak peaks appearing at 2θ = 33.3° and 39.8° [45].

In the case of both empty CS-PLGA NPs and Bupi-CS-PLGA NPs, a broad diffraction pattern was observed with a prominent diffraction peak corresponding to a polymers-related one at approximately 2θ = 19–20°. It should be noted that the distinct crystallographic reflections which can be seen in the diffraction pattern of pure Bupi-HCl cannot be distinguished in the diffraction pattern of Bupi-CS-PLGA NPs. Both samples displayed comparable diffraction patterns with small variations in terms of the breadth and intensity of the broad peaks.

#### 3.1.4. Encapsulation Efficiency (EE) and Drug Loading (DL)

**Figure 5:**
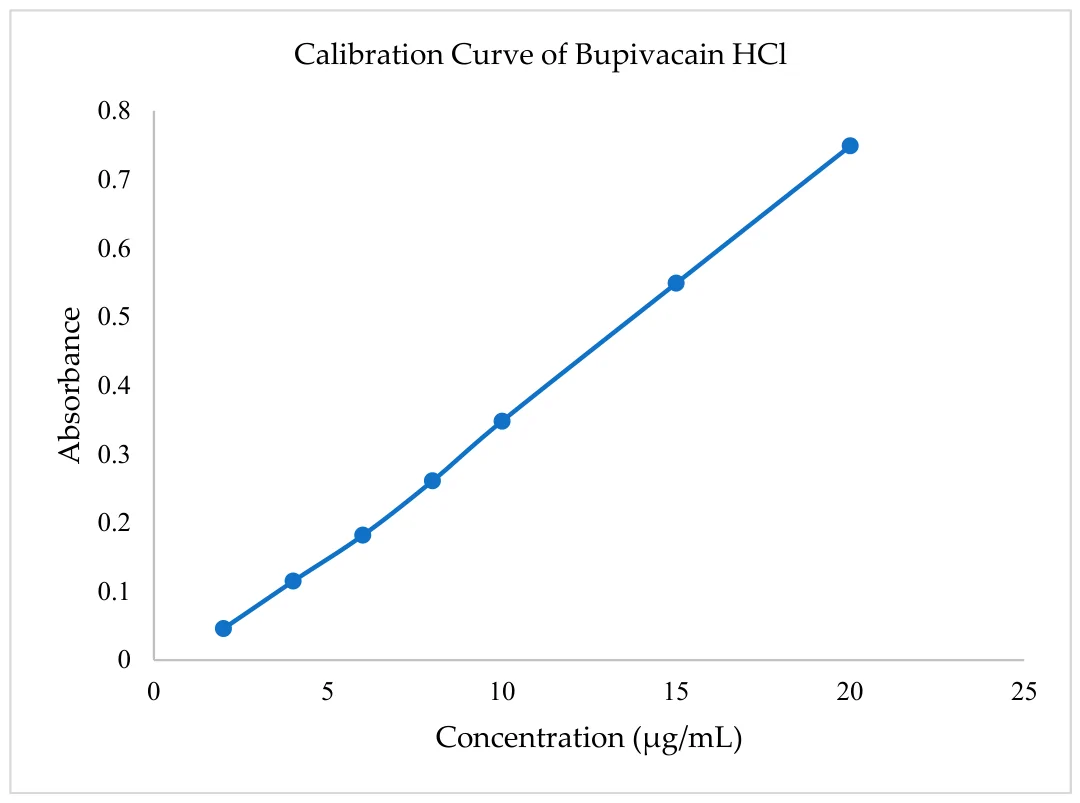
Calibration curve of Bupi-HCl determined by UV-Visible spectrophotometry at 201 nm over the concentration range of 2–20 µg/mL.

The quantitative determination of Bupi-HCl was performed by measuring the absorbances of standard solutions within the concentration range of 2–20 µg/mL at 201 nm, followed by the construction of a calibration curve (Figure 5).

Linear regression analysis yielded the equation y = 0.0395x − 0.0453 with an R^2^ value of 0.999, demonstrating good linearity over the concentration range of 2–20 µg/mL.

The calibration curve was used to calculate the amount of free Bupi-HCl in the supernatant. Based on these values, the encapsulation efficiency and drug loading capacity of the nanoparticles were determined (Table 2).

The Bupi-CS-PLGA NPs exhibited an encapsulation efficiency of 80.00 ± 2.14% and a drug-loading capacity of 2.60 ± 0.11% (Table 2).

### 3.2. In Vitro Drug Release Study

**Figure 6:**
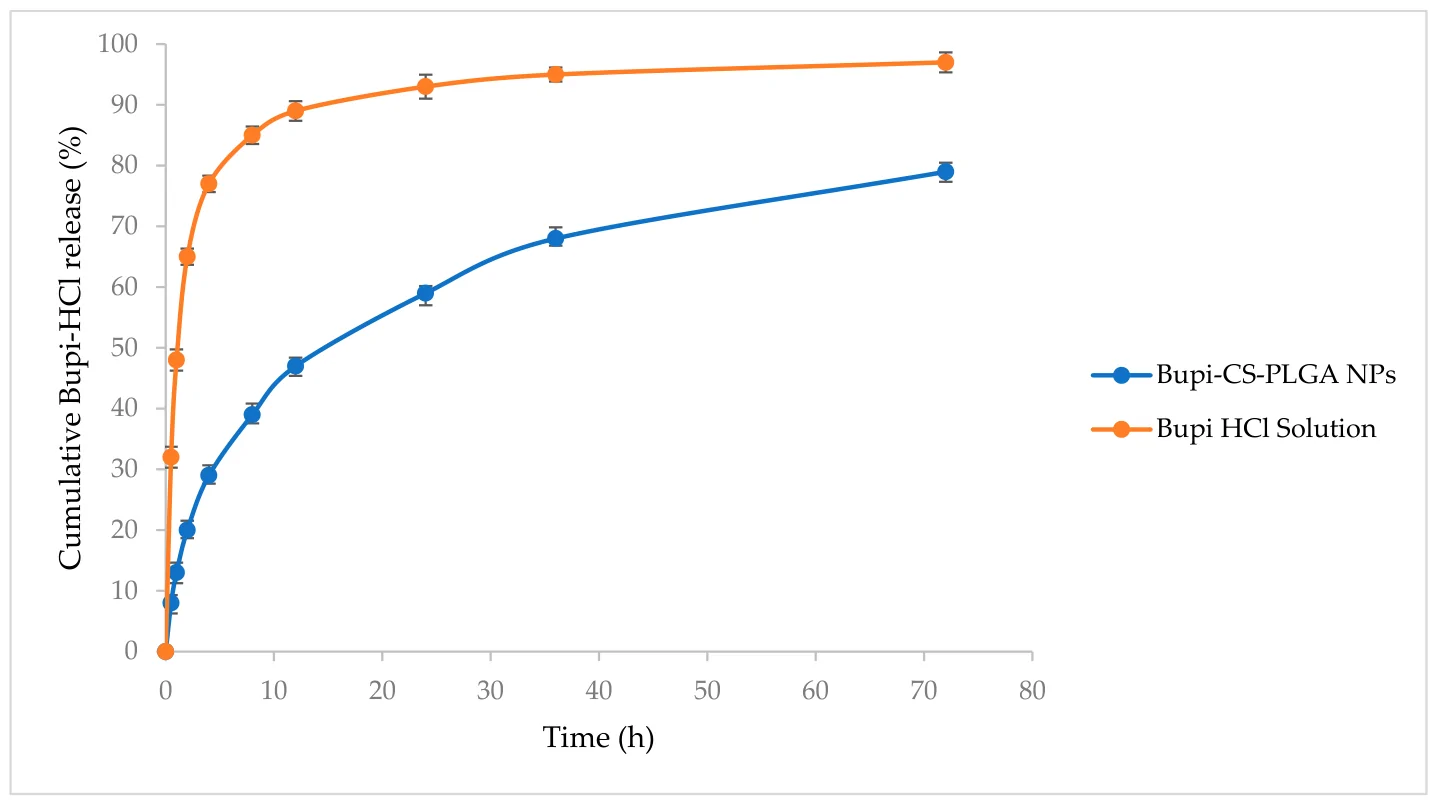
In vitro cumulative drug release profiles of Bupi-CS-PLGA NPs and free Bupi-HCl solution. Data are presented as mean ± SD (n = 3).

Cumulative release profiles obtained from the in vitro release study using Franz diffusion cells are presented in Figure 6. Distinct differences were observed between the release profiles of free Bupi-HCl and Bupi-CS-PLGA NPs. The free Bupi-HCl solution exhibited a rapid release profile from the initial hours of the study. About 77% of the drug was released within 4 h, and cumulative release reached 85% by the 8th hour. The amount of drug released was around 89% by the 12th hour, 93% by the 24th hour, and 95% by the 36th hour. By the 72nd hour, about 97–98% of the drug was released. Bupi-CS-PLGA nanoparticles exhibited a much slower release profile than the free drug. Around 30% release was observed within the first 4 h, followed by cumulative releases of around 39% and 47% at the 8th and 12th hours, respectively. By 24 h, around 59% release was achieved, followed by 68% by the 36th hour and around 79–80% by the end of 72 h.

The free Bupi-HCl solution exhibited rapid release, whereas Bupi-CS-PLGA NPs showed a sustained release profile throughout the study period. Bupi-CS-PLGA NPs exhibited a markedly slower release profile than free Bupi-HCl, particularly during the first 24 h, with approximately 79–80% cumulative release at 72 h. The release data were fitted to nine mathematical models, and the corresponding R^2^ and total residual values are presented in Table 3. Among the evaluated models, Modified Langmuir and RRSBW showed the highest coefficients of determination (R^2^ = 0.9958 and 0.9952, respectively), followed by BTa (R^2^ = 0.9901). RRSBW exhibited the lowest total residual value (19.5851), followed by Modified Langmuir (42.9845) and BTa (54.2297). The remaining models showed lower coefficients of determination and/or higher residual values (Table 3). Thus, the experimental release profile was well described by more than one mathematical model, and no single model was selected solely on the basis of R^2^.

### 3.3. Cytocompatibility Evaluation

**Figure 7:**
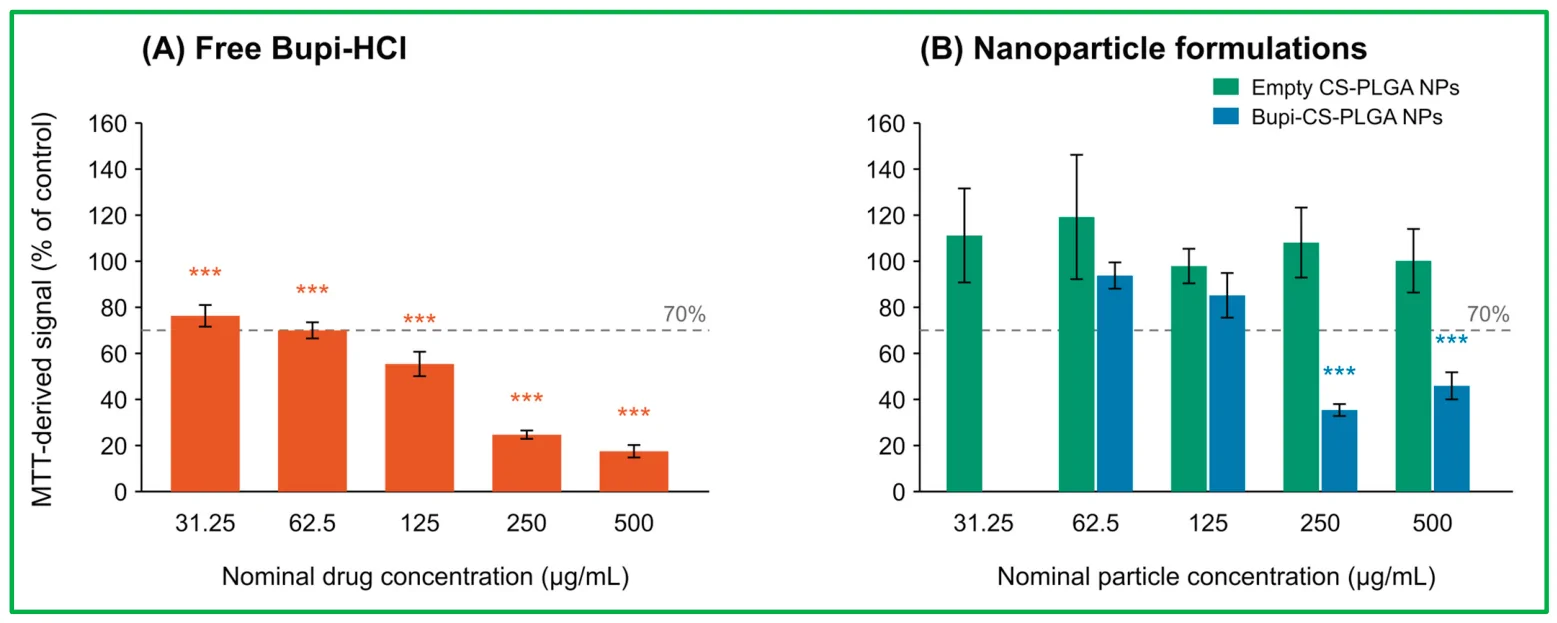
MTT-derived signal of HDFa fibroblasts after 48 h of exposure. (**A**) Free Bupi-HCl, with the concentration axis expressed as drug mass. (**B**) Empty CS-PLGA NPs and Bupi-CS-PLGA NPs, with the concentration axis expressed as total particle mass; the two panels are therefore not directly comparable as potency measures. In both panels the dashed line marks the 70% viability level, which corresponds to the cytotoxicity reference threshold of ISO 10993-5 [46] and is shown only as a descriptive reference line, not as a formal ISO conformity determination. Bars show mean ± SD of six technical replicate wells from one independent experiment; the loaded formulation was not tested at 31.25 µg/mL, and the free drug and nanoparticle formulations were tested on separate plates. Asterisks denote exploratory within-run Holm-adjusted Welch’s *t*-tests versus the untreated control on the same plate (*** *p* < 0.001); comparisons without a marker were not significant. The MTT readout was not confirmed with an orthogonal viability endpoint, and particle-specific interference was not excluded.

A preliminary cytocompatibility assessment of free Bupi-HCl, empty CS-PLGA NPs, and Bupi-CS-PLGA NPs was performed in HDFa cells using the MTT assay after 48 h of exposure (Figure 7; Table 4). Results were expressed as MTT-derived viability relative to the corresponding untreated control.

#### 3.3.1. Effect of Empty CS-PLGA NPs

Empty CS-PLGA NPs yielded MTT-derived signals around the untreated-control level across the investigated concentration range. MTT-derived values were 111.2 ± 20.4%, 119.2 ± 27.0%, 97.9 ± 7.5%, 108.1 ± 15.2%, and 100.2 ± 13.8% at nominal particle concentrations of 31.25, 62.5, 125, 250, and 500 µg/mL, respectively. No reduction from the corresponding untreated control was detected by exploratory within-run comparisons (Holm-adjusted *p* > 0.05). This single-run result does not by itself establish biological cytocompatibility.

#### 3.3.2. Effect of Free Bupi-HCl

Free Bupi-HCl produced a concentration-dependent reduction in MTT-derived viability following 48 h of exposure. Viability was 76.3 ± 4.7%, 70.0 ± 3.5%, 55.4 ± 5.3%, 24.7 ± 1.8%, and 17.5 ± 2.7% at 31.25, 62.5, 125, 250, and 500 µg/mL, respectively. Within this single experimental run, all tested free Bupi-HCl concentrations showed lower MTT-derived viability than the corresponding untreated control (Holm-adjusted *p* < 0.001).

#### 3.3.3. Effect of Bupi-CS-PLGA NPs

Bupi-CS-PLGA NPs yielded MTT-derived signals of 93.8 ± 5.7% and 85.2 ± 9.7% at nominal particle concentrations of 62.5 and 125 µg/mL, respectively. Neither concentration differed from the corresponding untreated control within this single run (Holm-adjusted *p* = 0.280 and *p* = 0.077, respectively).

At higher particle concentrations, MTT-derived signal was 35.4 ± 2.6% at 250 µg/mL and 45.9 ± 5.9% at 500 µg/mL, with both values lower than the corresponding untreated control within this single run (Holm-adjusted *p* < 0.001). The MTT-derived signal at 500 µg/mL was higher than at 250 µg/mL; this non-monotonic pattern was observed across the technical replicate wells and was not attributable to an identifiable outlier. At matched nominal particle concentrations, exploratory statistical comparisons indicated lower MTT-derived signal for Bupi-CS-PLGA NPs than for empty CS-PLGA NPs at 250 and 500 µg/mL (Holm-adjusted *p* < 0.001), whereas no differences were observed at 62.5 and 125 µg/mL (Holm-adjusted *p* = 0.070 and *p* = 0.062, respectively). Based on the experimentally determined drug loading of 2.60% (*w*/*w*), Bupi-CS-PLGA NP concentrations of 62.5, 125, 250, and 500 µg/mL corresponded to nominal Bupi-HCl-equivalent concentrations of 1.63, 3.25, 6.50, and 13.0 µg/mL, respectively. These drug-equivalent concentrations were below the lowest directly tested free Bupi-HCl concentration of 31.25 µg/mL. Because dose-equivalent concentrations of free Bupi-HCl were not evaluated, the present data do not permit a direct quantitative comparison of cytotoxic potency between free Bupi-HCl and Bupi-CS-PLGA NPs. Table 4 summarizes the MTT-derived results and corresponding within-run statistical comparisons.

Free Bupi-HCl concentrations represent drug mass, whereas Bupi-CS-PLGA NP concentrations represent total nanoparticle mass. Because dose-equivalent concentrations of free and encapsulated Bupi-HCl were not evaluated, these data should be interpreted descriptively; rows with the same nominal mass concentration do not constitute drug-equivalent comparisons between formulations.

### 3.4. DPPH Assay

**Figure 8:**
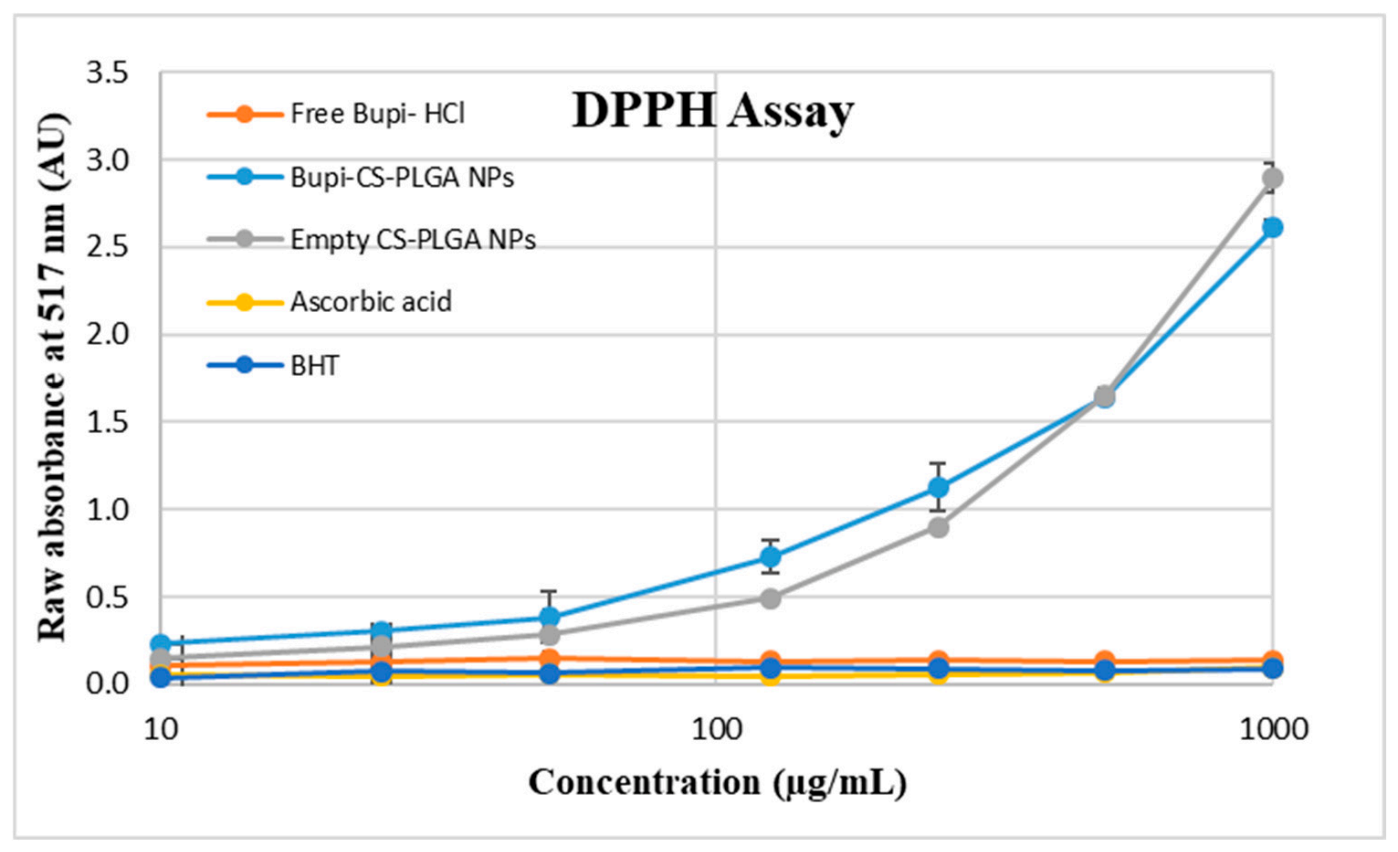
Raw absorbance at 517 nm of free Bupi-HCl, empty CS-PLGA NPs, Bupi-CS-PLGA NPs, ascorbic acid, and BHT in the DPPH assay at nominal concentrations of 10–1000 µg/mL. Points represent means, and error bars indicate SD (three wells per concentration). The concentration axis is logarithmic. Sample-specific blanks without DPPH were not measured; therefore, the nanoparticle absorbance profiles should not be interpreted as quantitative radical-scavenging activity.

The raw absorbance at 517 nm of free Bupi-HCl ranged from 0.1057 ± 0.0006 at 10 µg/mL to 0.1450 ± 0.0122 at 50 µg/mL. At 10 µg/mL, the absorbance values of empty CS-PLGA NPs and Bupi-CS-PLGA NPs were 0.1450 ± 0.0036 and 0.2323 ± 0.0085, respectively. At 1000 µg/mL, the corresponding values were 2.8923 ± 0.0836 and 2.6110 ± 0.0407, respectively. The mean raw absorbance of the DPPH-plus-solvent control wells was 0.1281 (21 wells). The absorbance values at each concentration are reported in Table 5 and plotted in Figure 8.

### 3.5. Stability Study

The physicochemical stability of Bupi-CS-PLGA nanoparticles was evaluated during storage for 12 months at 25 ± 2 °C/60 ± 5% relative humidity and under refrigerated conditions (5 ± 2 °C) (Table 6 and Table 7).

## 4. Discussion

The physicochemical properties of polymeric nanoparticles dictate their colloidal stability, drug-release profile, and biological performance [47,48]. In the present study, Bupi-CS-PLGA NPs were roughly spherical, with a smooth surface and a narrow particle-size distribution. Spherical nanoparticles have lower surface free energy than irregular ones and therefore have a lower tendency to agglomerate and better physical stability during storage [49]. TEM images supported this observation, showing predominantly dispersed particles with limited particle contact. These observations are consistent with the formation of a relatively homogeneous nanoparticle dispersion [30]. Similar morphological features have been reported for CS-PLGA NPs prepared by similar fabrication methods. Drug loading caused only a slight increase in hydrodynamic diameter while maintaining a PDI below 0.20, indicating that incorporating Bupi-HCl did not compromise formulation homogeneity [50]. The increase in particle size is expected following encapsulation and chitosan coating because both processes increase the hydrated diameter measured by dynamic light scattering [51,52]. A PDI below 0.20 indicates a relatively narrow and homogeneous particle-size distribution [50]. Similar results were recently reported for chitosan-coated PLGA nanoparticles displaying narrow size distribution and good colloidal stability. The observed positive zeta potential after nanoparticle preparation indirectly indicates successful chitosan coating [53,54]. Native PLGA nanoparticles are usually negatively charged due to the terminal carboxyl groups on their surface [55]. The surface charge is positive after adsorption of protonated amino groups from chitosan [56]. The positive zeta potential of both empty and Bupi-HCl-loaded nanoparticles was consistent with successful chitosan surface modification, while the similar zeta-potential values before and after drug loading suggested that incorporation of Bupi-HCl did not markedly alter the overall surface charge. Such changes in zeta potential have been reported for PLGA nanoparticles after chitosan surface modification and are often associated with enhanced colloidal stability and surface functionality [57,58]. The good agreement between the TEM and the DLS measurements also supports the physicochemical characterization of the above-mentioned systems. As expected, the particle sizes obtained by DLS were somewhat larger than those measured by TEM, because DLS measures the hydrodynamic diameter of hydrated particles in suspension whereas TEM measures the dry particle core under vacuum. Such differences are common for polymeric nanoparticles and should be considered as complementary rather than contradictory [59,60]. These physicochemical properties of the prepared Bupi-CS-PLGA NPs suggest that they may serve as a stable platform for sustained drug delivery and further biological evaluation.

Overall, the present findings demonstrate favorable physicochemical characteristics, sustained drug release, and long-term colloidal stability, together with preliminary data on the fibroblast cytocompatibility of the Bupi-CS-PLGA NPs. Possible molecular interactions among Bupi-HCl, PLGA, and chitosan were investigated using FTIR analysis. Characteristic absorption bands of PLGA and chitosan were retained even after the preparation of the nanoparticles, and no additional bands were observed for the drug-loaded formulation. The characteristic ester carbonyl band of PLGA and the characteristic hydroxyl and amino-related bands of chitosan were retained in the nanoparticle spectra, supporting the presence of both polymers in the formulated system and suggesting that no major alteration of their characteristic functional groups occurred during nanoparticle preparation [61,62].

No new absorption bands were observed, although slight decreases in the intensity of several characteristic Bupi-HCl peaks and minor band shifts were detected following encapsulation. Such spectral changes, in the absence of new absorption bands, may reflect weak non-covalent interactions between the drug and the polymeric matrix, including hydrogen bonding, rather than the formation of new covalent bonds [63,64]. The functional groups of PLGA (ester carbonyl groups) and chitosan (hydroxyl and protonated amino groups) can interact with the entrapped drug without affecting its chemical structure. Thus, the observed spectral changes are consistent with physical entrapment of Bupi-HCl in the polymer matrix, not chemical conjugation.

Preservation of the characteristic FTIR bands is particularly important because it suggests that nanoparticle preparation did not modify the chemical structure of either the drug or the carrier polymers [65]. Similar observations have recently been reported for CS-PLGA-NPs encapsulating various therapeutic agents, where retention of characteristic absorption bands, along with only minor peak shifts, was interpreted as evidence of successful physical encapsulation rather than chemical interaction [66,67].

FTIR results should not, however, be interpreted in isolation. Although the technique confirms the absence of significant chemical incompatibility, it cannot distinguish whether the encapsulated drug remains crystalline or becomes molecularly dispersed within the polymer matrix. Therefore, FTIR data should be supplemented with other solid-state characterization techniques. The FTIR findings, considered together with the complementary DSC and XRD results, were consistent with the incorporation of Bupi-HCl into the PLGA/chitosan nanoparticle system without evidence of new chemical bond formation.

The DSC profiles of the empty and Bupi-HCl-loaded nanoparticles differed from those of the individual polymeric components, with changes in the position, intensity, and breadth of the observed thermal events. Such alterations in thermal behavior may occur following nanoparticle formation as a consequence of changes in polymer-chain organization, solvent evaporation, particle solidification, and incorporation of the drug into the polymeric matrix [63,68,69,70]. The high-temperature thermal event observed for chitosan near 330 °C was consistent with its previously reported thermal degradation behavior [44]. However, because pure Bupi-HCl was not analyzed separately by DSC in the present study, specific thermal events in the Bupi-CS-PLGA NP thermogram cannot be directly assigned to Bupi-HCl. Therefore, the DSC findings alone do not allow conclusions regarding the disappearance of a characteristic Bupi-HCl melting event or a change in the crystalline state of the drug. Accordingly, the solid-state detectability of Bupi-HCl within the nanoparticle formulation was evaluated primarily on the basis of the complementary XRD findings.

X-ray diffraction analysis confirmed the crystalline nature of pure Bupi-HCl, which showed several sharp and intense diffraction reflections. Several of the major experimental reflections observed around 2θ = 9–10°, 19°, and 25° were consistent with previously reported diffraction features of crystalline bupivacaine [43]. Conversely, PLGA exhibited mainly broad diffraction profile in consistence with its predominantly amorphous nature. This is in agreement with the previously reported XRD features of PLGA based systems [70,71]. Chitosan showed characteristic diffraction features, including a prominent reflection around 2θ = 20°, consistent with previously reported chitosan diffraction behavior and the reported JCPDS No. 39-1894 reference pattern [44]. The typical crystalline reflections of pure Bupi-HCl were not observed as sharp peaks in the Bupi-CS-PLGA NP formulation indicating that Bupi-HCl was not detected as a separate crystalline phase in the PLGA/chitosan matrix at the experimental XRD conditions. Similar findings have been reported for drug-loaded PLGA and PLGA–chitosan nanoparticles, in which characteristic drug-related crystalline reflections were markedly reduced or no longer detectable following incorporation into the polymeric matrix [70,71,72]. However, the absence of detectable Bupi-HCl reflections should not be interpreted as definitive evidence of complete drug amorphization, because the relatively low drug loading and dispersion of Bupi-HCl within the polymeric matrix may also reduce the detectability of crystalline drug reflections by XRD. Collectively, these results supported the physical entrapment of Bupi-HCl in PLGA/chitosan matrix. The XRD analysis showed that there was no obvious crystalline Bupi-HCl phase in the nanoparticle formulation.

Drug loading caused only minor changes in the width and intensity of the amorphous halo, suggesting that Bupi-HCl incorporation did not markedly affect the predominantly amorphous characteristics of the PLGA/chitosan matrix [73]. The sustained-release behavior observed in the present study should therefore be considered primarily in relation to drug incorporation within the polymeric matrix and the presence of the chitosan coating rather than being attributed to changes in drug crystallinity alone [11,13]. These findings are consistent with recent studies reporting sustained drug release from PLGA/chitosan nanoparticle systems [74].

The high EE achieved in this study indicates that the modified double-emulsion solvent-evaporation technique effectively retained Bupi-HCl within the PLGA matrix despite its hydrophilic nature. Encapsulation of hydrophilic compounds in PLGA nanoparticles is generally challenging because of their tendency to partition from the internal phase into the external aqueous phase during emulsification, resulting in drug loss and reduced encapsulation efficiency [28,75]. Nevertheless, high encapsulation efficiencies have been achieved for hydrophilic compounds by optimizing double-emulsion-based PLGA nanoparticle preparation conditions [75,76]. Accordingly, the high EE obtained in the present study suggests that the selected formulation composition and processing conditions effectively limited Bupi-HCl loss during nanoparticle preparation. Although the drug loading was relatively low, this is consistent with the relatively high polymer-to-drug ratio used in the present formulation, as drug-to-polymer ratio is an important determinant of the drug-loading capacity of PLGA nanoparticles [77].

The in vitro release study showed that encapsulation of Bupi-HCl in chitosan-coated PLGA nanoparticles markedly altered the release profile compared with that of the free drug solution. These findings demonstrate that incorporation into the polymeric carrier markedly modified the Bupi-HCl release profile and provided sustained drug release over the investigated 72 h period. The sustained-release profile may be influenced by the structural characteristics of the PLGA matrix and the presence of the chitosan coating [11,13]. The observed release behavior is consistent with previous reports suggesting that drug diffusion through hydrated polymeric matrices can contribute to sustained release from PLGA-based systems [11,78]. Chitosan coating has also been reported to provide an additional diffusional barrier in PLGA-based delivery systems [79]. However, polymer degradation and individual release mechanisms were not directly measured in the present study; therefore, the kinetic findings should be interpreted as mathematical descriptors of the release profile rather than definitive evidence of a specific release mechanism. The rapid diffusion of free Bupi-HCl may cause high local drug concentrations immediately after administration and a relatively short duration of action; therefore, minimizing the initial burst release is beneficial for sustained local anesthetic delivery. Sustained-release nanoparticle systems may modulate local drug availability and free-drug exposure. However, the present cytocompatibility experiment was not designed to directly compare the cytotoxic effects of free and encapsulated Bupi-HCl at equivalent drug concentrations. Therefore, the MTT findings should be interpreted as a preliminary assessment of the cytocompatibility of the nanoparticle formulation rather than as a direct comparison with free Bupi-HCl.

The release profile of the Bupi-CS-PLGA NPs was assessed with nine kinetic models using mathematical analysis. The Modified Langmuir model and the RRSBW had almost similar coefficients of determination (R^2^) (0.9958 and 0.9952, respectively), while BTa model gave a high R^2^ value (0.9901). However, the RRSBW model gave the lowest total residual value (19.5851), compared to 42.9845 for Modified Langmuir and 54.2297 for BTa model. These findings demonstrate that assessment based solely on R^2^ would not fully reflect the differences among the fitted models, because the model with the highest R^2^ was not the model with the lowest residual value. Therefore, rather than assigning a single “best-fitting” model based exclusively on R^2^, the kinetic analysis was interpreted as a comparative mathematical description of the experimental release profile. Although the Higuchi model showed a relatively high coefficient of determination (R^2^ = 0.9509), model fitting alone does not establish a unique physicochemical release mechanism. Accordingly, diffusion may contribute to Bupi-HCl release from the nanoparticle matrix, but the present kinetic analysis does not demonstrate diffusion as the sole release mechanism. In the context of previous bupivacaine-loaded PLGA and chitosan-containing delivery systems discussed above, the pharmaceutical relevance of the present Bupi-CS-PLGA formulation lies in the integration of a biodegradable PLGA matrix with a cationic chitosan coating and its comprehensive evaluation in terms of physicochemical and solid-state properties, 72 h sustained release and release kinetics, 12-month physicochemical stability, and preliminary cytocompatibility in primary adult human dermal fibroblasts. While liposomes containing bupivacaine serve as a clinically proven option for prolonging local anesthesia, biodegradable polymeric nanoparticles constitute a different approach to formulation development, where release profiles of the drug can be tuned using a polymer and surface modification [19]. It should be noted that this study does not prove the clinical advantage over other available options for delivering bupivacaine, but it has pharmaceutical significance since this system was characterized in terms of sustained Bupi-HCl release during 72 h and largely maintained physicochemical parameters during 12 months of storage. Long-term physicochemical stability is an important consideration for polymeric nanoparticle formulations intended for pharmaceutical applications [80,81].

In the present study, no marked changes in the measured physicochemical characteristics of Bupi-CS-PLGA NPs were observed during the 12-month storage period under either refrigerated or room-temperature conditions. Only minor changes were observed in particle size, while PDI and zeta potential remained largely unchanged. The consistently low PDI values and maintained positive surface charge indicate that the colloidal properties of the nanoparticle dispersion were preserved during storage. Similar stability assessments of polymeric and chitosan-based nanoparticles have used changes in particle size, PDI, and zeta potential as indicators of colloidal stability during storage [68,82].

Evaluation of fibroblast cytocompatibility is important for local-anesthetic delivery systems because bupivacaine and other local anesthetics can reduce fibroblast viability and proliferative/metabolic activity in a concentration-dependent manner [26,27]. Empty CS-PLGA nanoparticles yielded MTT-derived signals comparable to control across the investigated concentration range in this single experiment. Published evidence also indicates that the cytotoxicity of chitosan-modified PLGA nanoparticles depends on formulation properties, cell type, culture medium, and colloidal stability [58]. Comparable MTT-based cytocompatibility has been reported for chitosan-coated PLGA nanoparticles carrying benzocaine in 3T3 fibroblasts and HaCaT keratinocytes [25,46]; because that system differs in drug, cell type, and formulation, it provides analogous rather than direct support for the present HDFa findings. As expected, free Bupi-HCl decreased MTT-derived viability in a concentration-dependent manner, consistent with previous fibroblast studies of local-anesthetic cytotoxicity [26,27]. Bupi-CS-PLGA NPs maintained relatively high MTT-derived viability at 62.5 and 125 µg/mL, whereas a marked reduction in the MTT signal was observed at 250 and 500 µg/mL. The response was not monotonic between the two highest concentrations. The lower MTT-derived signal at 250 and 500 µg/mL cannot be attributed confidently to reduced cell survival: particle-specific interference was not assessed, and sustained release measured in a Franz-cell system does not establish cellular exposure in the culture well. The free-drug and nanoparticle formulations were evaluated across different concentration scales; therefore, a direct quantitative comparison of cytotoxic potency is not appropriate. The formulation’s sustained-release behavior provides a plausible basis for modulating free-drug exposure; however, the present cytocompatibility experiment was not designed to demonstrate reduced cellular exposure relative to an equivalent concentration of free Bupi-HCl. An additional limitation is that the cytocompatibility assessment was based on a single independent experiment with technical replicates. Therefore, the statistical findings should be considered exploratory and require confirmation in independent biological experiments using low-passage HDFa cells. The use of HDFa cells at passage 24 represents an additional limitation, as prolonged passaging may influence fibroblast proliferative and metabolic characteristics and therefore affect interpretation of MTT-derived viability. An additional methodological limitation is that cell-free wells containing the nanoparticle dispersions were not included as particle-specific optical controls. Therefore, nanoparticle-related interference with MTT reduction or absorbance detection cannot be completely ruled out, particularly at higher particle concentrations [83]. Furthermore, the MTT assay reflects cellular metabolic activity rather than directly measuring cell number or membrane integrity. Accordingly, definitive interpretation requires confirmation using independent biological experiments and an orthogonal viability endpoint. The preliminary MTT findings warrant further validation before fibroblast cytocompatibility can be concluded. The physicochemical characterization, EE/DL determinations, and stability assessment were based on replicate measurements of the prepared formulation rather than independently prepared batches; therefore, evaluation of independently prepared batches in future studies would further establish batch-to-batch reproducibility.

The DPPH assay is commonly used to assess chemical radical-scavenging capacity through electron- and/or hydrogen-donation mechanisms, with a decrease in DPPH absorbance indicating radical reduction when sample-associated optical contributions are adequately controlled [84,85,86]. In the present study, ascorbic acid and BHT exhibited lower raw absorbance values than the test formulations across the nominal concentration range of 10–1000 µg/mL. However, because concentration-matched sample blanks without DPPH were not measured, these differences should not be interpreted as a definitive quantitative comparison of antioxidant activity, particularly for the nanoparticle dispersions.

At 10 µg/mL, the raw absorbance values of free Bupi-HCl, empty CS-PLGA NPs, and Bupi-CS-PLGA NPs were 0.1057 ± 0.0006, 0.1450 ± 0.0036, and 0.2323 ± 0.0085, respectively. Although free Bupi-HCl showed its lowest raw absorbance at this concentration, this observation alone does not establish antioxidant activity. Both nanoparticle formulations exhibited increasing absorbance with increasing concentration, reaching 2.8923 ± 0.0836 for empty CS-PLGA NPs and 2.6110 ± 0.0407 for Bupi-CS-PLGA NPs at 1000 µg/mL. However, concentration-matched sample blanks without DPPH were not measured, preventing separation of formulation-associated background absorbance and light scattering from the DPPH signal.

Accordingly, these measurements are presented as exploratory raw absorbance profiles rather than quantitative estimates of radical-scavenging activity. The available data cannot establish whether nanoencapsulation altered the intrinsic antioxidant activity of Bupi-HCl. The comparatively preserved MTT-derived signal observed for Bupi-CS-PLGA NPs at lower particle concentrations therefore cannot be attributed to an antioxidant effect on the basis of these DPPH measurements. Encapsulation and sustained release may influence free-drug exposure, but their contribution to the observed cellular response remains a hypothesis that requires further investigation.

## 5. Conclusions

Bupi-CS-PLGA NPs were successfully prepared and comprehensively characterized. The nanoparticles exhibited a homogeneous spherical morphology, a narrow size distribution, a positive surface charge, and efficient drug encapsulation. Furthermore, they maintained their physicochemical characteristics throughout a 12-month storage period. Physicochemical characterization studies supported the finding that Bupi-HCl was physically incorporated into the PLGA/chitosan matrix; no evidence of new chemical bond formation was observed. XRD analysis indicated that no distinct crystalline Bupi-HCl phase could be detected in the nanoparticle formulation under the experimental conditions. Compared with free Bupi-HCl, the nanoparticle formulation provided sustained drug release over 72 h. Bupi-CS-PLGA NPs showed relatively high MTT-derived signals at 62.5 and 125 µg/mL and lower signals at 250 and 500 µg/mL, whereas empty CS-PLGA NPs yielded signals near the untreated-control level across the tested range. The high-concentration signals cannot be interpreted as definitive cell loss without particle-specific interference controls and an independent viability endpoint. However, since free and encapsulated Bupi-HCl were not evaluated at dose-equivalent concentrations, these findings should not be interpreted as evidence of reduced Bupi-HCl cytotoxicity following encapsulation. The kinetic study provided a mathematical description of the release profile. Modified Langmuir and RRSBW demonstrated similarly high coefficients of determination (R^2^ = 0.9958 and 0.9952, respectively), whereas RRSBW showed the lowest total residual value among all evaluated mathematical models. These findings indicate that the experimental release profile can be well described by several mathematical models; therefore, the kinetic results were interpreted as mathematical descriptors rather than as definitive evidence of a specific release mechanism. Overall, the combined physicochemical characteristics, sustained-release behavior, long-term physicochemical stability, and preliminary, assay-limited fibroblast MTT observations motivate further evaluation of Bupi-CS-PLGA NPs as a biodegradable carrier for sustained Bupi-HCl delivery. Future studies should include dose-equivalent comparisons with free Bupi-HCl and confirmation of the preliminary MTT findings in independent biological experiments using complementary viability endpoints, followed by in vivo pharmacokinetic, efficacy, and safety evaluations.

## Figures and Tables

**Table 1:** Physicochemical characteristics of empty CS-PLGA NPs and Bupi-CS-PLGA NPs. Data are presented as mean ± SD of 10 replicate measurements performed on samples obtained from the same prepared formulation (n = 10).

Formulations	Particle Size (nm)	PDI	Zeta Potential (mV)
CS-PLGA NPs	161.150 ± 5.601	0.148 ± 0.012	+24.104 ± 1.021
Bupi-CS-PLGA NPs	175.132 ± 8.103	0.195 ± 0.026	+24.861 ± 1.90

**Table 2:** Encapsulation efficiency (EE) and drug loading (DL) of Bupi-CS-PLGA NPs. Data are presented as mean ± SD of three analytical replicate measurements performed on samples obtained from the same prepared formulation (n = 3).

Formulation	EE	DL
Bupi-CS-PLGA NPs	80.00 ± 2.14	2.60 ± 0.11

**Table 3:** Mathematical model fitting of the in vitro release profile of Bupi-CS-PLGA nanoparticles.

Model	R^2^	Total Residual
RRSBW	0.9952	19.5851
Modified Langmuir	0.9958	42.9845
(*Bt*)^*a*^	0.9901	54.2297
Higuchi	0.9509	245.0940
Langmuir	0.9595	385.5068
First-Order	0.9242	543.1987
Hopfenberg	0.7875	1060.2840
Hixson–Crowell	0.8830	3794.2220
Zero-Order	0.7875	6066.6144

**Table 4:** MTT-derived signal of HDFa fibroblasts after 48 h exposure (%, mean ± SD of six technical replicate wells from one independent experiment; expressed as a percentage of the untreated control on the same plate). Free Bupi-HCl concentrations denote drug mass; nanoparticle concentrations denote total particle mass. Reported *p*-values are Holm-adjusted (Welch’s *t*-test versus the untreated control on the same plate).

Nominal Mass (µg/mL)	Nominal Bupivacaine Equivalent of Loaded NPs (µg/mL)	Free Bupi-HCl	Holm-Adjusted *p*	Bupi-CS-PLGA	Holm-Adjusted *p*	Empty CS-PLGA NPs
500	13.0	17.5 ± 2.7	<0.001	45.9 ± 5.9	<0.001	100.2 ± 13.8
250	6.50	24.7 ± 1.8	<0.001	35.4 ± 2.6	<0.001	108.1 ± 15.2
125	3.25	55.4 ± 5.3	<0.001	85.2 ± 9.7	0.077	97.9 ± 7.5
62.5	1.63	70.0 ± 3.5	<0.001	93.8 ± 5.7	0.280	119.2 ± 27.0
31.25	—	76.3 ± 4.7	<0.001	—	—	111.2 ± 20.4

**Table 5:** Raw absorbance at 517 nm of free Bupi-HCl, empty CS-PLGA NPs, Bupi-CS-PLGA NPs, ascorbic acid, and BHT in the DPPH assay. Values are presented as mean ± SD of three wells per concentration. Concentration-matched sample blanks without DPPH were not measured; therefore, the nanoparticle absorbance profiles should not be interpreted as quantitative DPPH radical-scavenging activity.

Conc. (µg/mL)	Free Bupi-HCl	Bupi-CS-PLGA NPs	Empty CS-PLGA NPs	Ascorbic Acid	BHT
10	0.1057 ± 0.0006	0.2323 ± 0.0085	0.1450 ± 0.0036	0.0553 ± 0.0075	0.0383 ± 0.0029
25	0.1257 ± 0.0067	0.3040 ± 0.0135	0.2133 ± 0.0179	0.0460 ± 0.0040	0.0757 ± 0.0101
50	0.1450 ± 0.0122	0.3837 ± 0.1435	0.2853 ± 0.0137	0.0563 ± 0.0133	0.0640 ± 0.0087
125	0.1313 ± 0.0050	0.7290 ± 0.0960	0.4947 ± 0.0093	0.0487 ± 0.0075	0.0943 ± 0.0084
250	0.1343 ± 0.0031	1.1270 ± 0.1337	0.9010 ± 0.0211	0.0603 ± 0.0155	0.0890 ± 0.0125
500	0.1303 ± 0.0125	1.6420 ± 0.0147	1.6517 ± 0.0405	0.0683 ± 0.0006	0.0787 ± 0.0065
1000	0.1363 ± 0.0175	2.6110 ± 0.0407	2.8923 ± 0.0836	0.0930 ± 0.0201	0.0887 ± 0.0214

**Table 6:** Stability test results of Bupi-CS-PLGA NPs stored at 25 ± 2 °C/60 ± 5% relative humidity. Data are presented as mean ± SD of 10 replicate measurements performed on samples obtained from the same stored formulation at each time point (n = 10).

	t = 0	t = 6 Months	t = 12 Months
Particle Size (nm)	175.132 ± 8.103	177.183 ± 5.027	185.203 ± 6.312
PDI	0.195 ± 0.026	0.196 ± 0.093	0.199 ± 0.059
Zeta potential (mV)	+24.861 ± 1.900	+24.414 ± 1.750	+24.351 ± 1.380

**Table 7:** Stability test results of Bupi-CS-PLGA NPs stored under refrigerated conditions (5 ± 2 °C). Data are presented as mean ± SD of 10 replicate measurements performed on samples obtained from the same stored formulation at each time point (n = 10).

	t = 0	t = 6 Months	t = 12 Months
Particle Size (nm)	175.132 ± 8.103	180.093 ± 4.216	180.105 ± 5.171
PDI	0.195 ± 0.026	0.195 ± 0.041	0.195 ± 0.097
Zeta potential (mV)	+24.861 ± 1.90	+24.698 ± 1.802	+24.912 ± 1.915

## Data Availability

The original contributions presented in this study are included in the article. Further inquiries can be directed to the corresponding authors.
